# The Rho guanosine nucleotide exchange factors Vav2 and Vav3 modulate epidermal stem cell function

**DOI:** 10.1038/s41388-022-02341-7

**Published:** 2022-05-09

**Authors:** L. Francisco Lorenzo-Martín, Mauricio Menacho-Márquez, Natalia Fernández-Parejo, Sonia Rodríguez-Fdez, Gloria Pascual, Antonio Abad, Piero Crespo, Mercedes Dosil, Salvador A. Benitah, Xosé R. Bustelo

**Affiliations:** 1 Molecular Mechanisms of Cancer Program, Centro de Investigación del Cáncer, Consejo Superior de Investigaciones Científicas (CSIC)-University of Salamanca, 37007 Salamanca, Spain; 2 Instituto de Biología Molecular y Celular del Cáncer, CSIC-University of Salamanca, 37007 Salamanca, Spain; 3 Centro de Investigación Biomédica en Red de Cáncer (CIBERONC), 37007 Salamanca, Spain; 4 Institut de Reserca Biomédica, 08028 Barcelona, Spain; 5 Instituto de Biomedicina y Biotecnología de Cantabria, CSIC-University of Cantabria, 39011 Santander, Spain

**Keywords:** Experimental organisms, Squamous cell carcinoma, RHO signalling, Cancer stem cells

## Abstract

It is known that Rho GTPases control different aspects of the biology of skin stem cells (SSCs). However, little information is available on the role of their upstream regulators under normal and tumorigenic conditions in this process. To address this issue, we have used here mouse models in which the activity of guanosine nucleotide exchange factors of the Vav subfamily has been manipulated using both gain- and loss-of-function strategies. These experiments indicate that Vav2 and Vav3 regulate the number, functional status, and responsiveness of hair follicle bulge stem cells. This is linked to gene expression programs related to the reinforcement of the identity and the quiescent state of normal SSCs. By contrast, in the case of cancer stem cells, they promote transcriptomal programs associated with the identity, activation state, and cytoskeletal remodeling. These results underscore the role of these Rho exchange factors in the regulation of normal and tumor epidermal stem cells.

## Introduction

The epidermis is one of the tissues with the highest cell turnover rates of the human body [1]. Mature skin cells are continuously sloughed into the environment and replaced by younger cells emerging from the basal layer of the epidermis [2]. This homeostatic balance is maintained by different populations of stem cells that reside in particular niches within the epidermis [3]. Among these, the cells located in the hair follicle bulge are considered the canonical SSCs [3–6]. This multipotent cell population has the potential to give rise to all epidermal lineages and structures, including the hair follicle, the sebaceous glands and the interfollicular epidermis [3, 5]. In line with this, it has been shown that the disruption of SSCs leads to several pathological conditions linked to wound repair, cancer, and ageing [7–9].

Rho GTPases are molecular switches that orchestrate a variety of cellular processes including cytoskeletal dynamics, polarity, migration, proliferation, and differentiation [10, 11]. Many of these processes are intimately associated with the homeostasis of SSCs [3]. In agreement with this, the genetic depletion of Rac1 leads to the differentiation of the SSC reservoir [12–14] and loss of clonogenic potential [12, 13, 15, 16]. In turn, RhoA contributes to the proliferation and migration of SSCs whereas Cdc42 regulates epidermal progenitor cell fate by controlling β-catenin turnover [17–19]. Despite this, very little is known up to now regarding the Rho GTPase modulators and effectors responsible for the engagement of these GTPase-dependent processes in vivo.

The activation of Rho GTPases is catalyzed by guanosine nucleotide exchange factors (GEFs), a group of enzymes that encompasses 83 members in mammalian species [20]. These enzymes promote the activation step of Rho proteins by mediating the release of the bound GDP from the inactive GTPases, thus favoring the subsequent GTP loading and the acquisition of the active, effector binding competent state [20]. Previous studies have shown that GEFs that activate Rac1 (e.g., Tiam1, Vav2, and Vav3) are important for skin carcinogenesis [21–23]. However, whether these or other GEFs are also involved in the physiological maintenance of SSCs is, to our knowledge, still unknown. Likewise, it is still unclear the contribution of these pathways to the functional status of tumor stem cell (TSCs). To address these issues, we have performed in this work in vivo, cell culture, genome-wide expression, and in silico analyses using both gain- and loss-of-function mouse models to establish the role of Vav2 and Vav3, the two exchange factors of the Vav family that are known to be implicated in skin tumorigenesis [23], in the biology of both SSCs and TSCs.

## Results

### Vav function is important for the regulation of epidermal stem cell numbers

We resorted to the use of genetically modified mouse models to study the contribution of Vav2 and Vav3 to the biological properties of bulge SSCs. To this end, we utilized on the one hand a previously described *Vav2*^Onc/Onc^ knock-in mouse model that expresses a gain-of-function version of Vav2 (Δ1–186, referred to hereafter as Vav2^Onc^). This mutant protein exhibits constitutive GEF activity due to the removal of two most N-terminal inhibitory domains (the calponin-homology [CH] and acidic [Ac] regions) [23–25]. This mouse model has some additional features that are important for the understanding of the experiments performed in this work: (i) The levels of the mutant Vav2^Onc^ protein found in the tissues of these mice are comparable to those exhibited by the wild-type (WT) counterpart, given that the mutant and WT alleles are expressed from the endogenous locus and are subjected to the same regulatory elements. Due to this, the mutant protein also shows the same cell type expression pattern that the wild-type counterpart [23–25]. These features ensure that, unlike the case of standard transgenic mice, the effects triggered by Vav2^Onc^ could not be attributed to the nonphysiological overexpression of the mutant protein or to the spurious induction of Vav2^Onc^ in cell lineages that usually do not exhibit Vav2^WT^ expression. (ii) The mutant protein keeps the adapter functions mediated by the C-terminal SH3–SH2–SH3 region intact [26, 27]. (iii) The mutant protein has lost the ability to stimulate the nuclear factor of stimulated T cells (NFAT) due to the elimination of the CH effector domain [26–29]. This feature, however, must not be relevant in the context of the skin given that this pathway is mostly B lymphocyte specific in the case of Vav2 [28]. Due to these features, the phenotypes observed can be exclusively linked to the constitutive stimulation of Vav2 catalytic activity rather than to changes in the adapter-like functions mediated the Vav2 C-terminal region [23–25]. Consistent with the idea that these mice exhibit a catalytic gain-of-function effect, we have found before that they show opposite phenotypes to those found in mice expressing a catalytically impaired Vav2 protein (L332A mutant) both in the skin and skeletal muscle [23, 25, 30, 31]. Likewise, they show alterations in cardiovascular homeostasis and skin tumorigenesis opposite to those exhibited by *Vav2*^–/–^ and *Vav2*^–/–^;*Vav3*^–/–^ knockout mice, respectively [21, 23, 24, 32, 33]. On the other hand, we used as loss-of-function models the previously described knockout mice for Vav2 and/or Vav3 [21, 34].

**Fig. 1:**
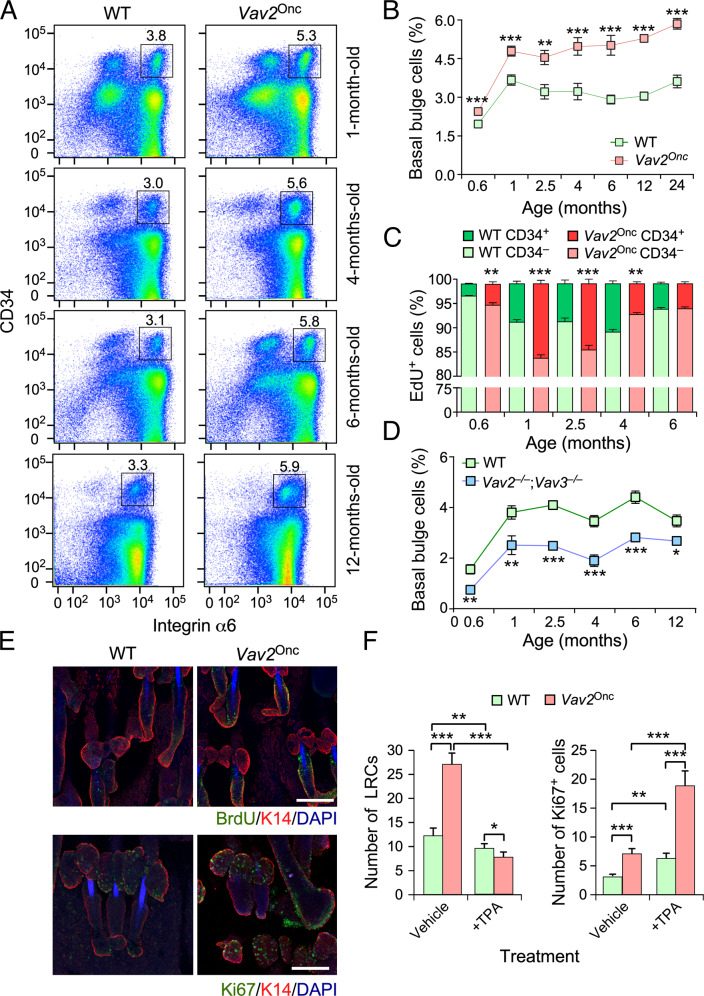
Vav function modulates epidermal stem cell numbers. **A** Representative flow cytometry dot plots from mice of indicated genotypes (top) and ages (right) showing the different epidermal cell populations according to CD34 and integrin α6 surface expression. The basal bulge population abundance is indicated (*n* = 6 per genotype and age). **B** Quantification of the abundance of CD34^+^ Itga6^+^ cells in the epidermis of mice of indicated genotypes and ages according to the data obtained in **A**. ***P < 0.001 (two-way ANOVA and Sidak’s multiple comparisons test, *n* as in **A**). **C** Quantification of the abundance of proliferating (EdU^+^) cells in the bulge (CD34^+^) of mice of the indicated genotypes and ages. **P < 0.01; ***P < 0.001 (two-way ANOVA and Sidak’s multiple comparisons test, *n* = 6 per genotype and time point). **D** Quantification of the abundance of CD34^+^ Itga6^+^ cells in the epidermis of mice of indicated genotypes. ***P < 0.001 (ANOVA and Dunnett’s multiple comparison test, *n* = 5). **E** Representative whole mount immunofluorescence images showing the presence of BrdU (green color, top panels) and the expression of Ki67 (green color, bottom panels) and keratin 14 (red color, all panels) in the tail epidermis from mice of indicated genotypes. DAPI (4’,6-diamidino-2-fenilindol) was used for counterstaining (blue color, all panels). Scale bar, 200 μm (*n* = 6). **F** Quantification of the number of label-retaining (left) and Ki67^+^ (right) cells in the hair follicle bulge of mice of indicated genotypes upon the treatments shown in **E**. *P < 0.05; **P < 0.01; ***P < 0.001 (ANOVA and Tukey’s HSD tests, *n* = 6). In **B**–**D** and **F**, data represent mean ± SEM.

To study whether the upregulation of Vav2 GEF activity could affect bulge SC homeostasis, we first analyzed the number of CD34^+^ Itgα6^+^ cells in the epidermis of *Vav2*^Onc/Onc^ mice by flow cytometry. Using this approach, we found that 8- to 10-week-old *Vav2*^Onc/Onc^ animals display increased numbers of SSCs when compared with WT animals (Fig. 1A, B). This expanded SSC population is subsequently conserved along the lifetime of those animals (Fig. 1B). We also observed using 5-ethynyl-2’-deoxyuridine (EdU) labeling studies that the germline expression of Vav2^Onc^ triggers SSC proliferation. However, this feature is restricted to animals younger than 4 months (Fig. 1C). In line with this elevated number of SSCs, Vav2^Onc^-expressing keratinocytes show increased clonogenic potential independently of the age of the animals tested when analyzed in cell culture (Fig. S1A, B).

To evaluate whether the function of Vav2^WT^ and/or Vav3^WT^ was also important for this process, we quantified the number of SSCs in single *Vav2*^–/–^, single *Vav3*^–/–^, and double *Vav2*^–/–^;*Vav3*^–/–^ knock-out animals. We could not detect any significant change in the numbers of CD34^+^ Itgα6^+^ SSCs in the single *Vav2*^–/–^ and *Vav3*^*–/–*^ knockout mice (Fig. S1C). By contrast, we did detect a decrease in SSC numbers in animals lacking both Vav2 and Vav3 (Fig. S1C). Consistent with these data, we found that the primary keratinocytes from *Vav2*^–/–^;*Vav3*^–/–^ mice, but not those from the single *Vav2*^–/–^ and *Vav3*^–/–^ animals, show reduced clonogenic activity when tested in cell culture (Fig. S1D). These two features are conserved throughout the lifetime of *Vav2*^*–/–*^;*Vav3*^*–/–*^ mice (Fig. S1E). These data indicate that the function of these two Vav family GEFs is important to ensure proper numbers of SSCs in mice.

### Vav function is important for epidermal stem cell responsiveness

One of the hallmarks of SSCs is their slow-cycling nature, a feature that makes them easily identifiable using 5-bromo-2’-deoxyuridine (BrdU)-retention studies [6]. We used this feature to further analyze the contribution of Vav proteins to SSC biology. To this end, we injected 10-day-old WT and *Vav2*^*Onc*^ mice with BrdU and, 80 days later, we quantified the number of BrdU^+^ and Ki67^+^ cells in the hair follicle bulge of the animals using whole mount immunofluorescence analyses. Consistent with the flow cytometry and EdU incorporation data (see above, Fig. 1A–C), we found that the hair follicle bulges from *Vav2*^Onc/Onc^ mice contain increased numbers of label-retaining (LRCs) (Fig. 1E, upper panels; Fig. 1F, left panel) and Ki67^+^ (Fig. 1E, lower panels; Fig. 1F, right panel) cells when compared to control animals. Conversely, the bulge samples from *Vav2*^*–/–*^;*Vav3*^*–/–*^ mice exhibit reduced numbers of LRCs (Fig. S1F, left panels; Fig. S1G, left panel; see vehicle bars). We could not observe, however, statistically significant changes in the very small number of Ki67^+^ cells that are present in the bulge of these animals (Fig. S1F, middle panels; Fig. S1G, right panel; see vehicle bars).

To assess the capacity of the SSCs to respond to external stimuli, we next subjected BrdU-labeled mice to topic applications of 12-O-tetradecanoylphorbol-13-acetate (TPA). This tumor promoter induces both the proliferation and mobilization of SSCs under normal conditions, leading to a reduction and an elevation in the number of LRCs and Ki67^+^ SSCs, respectively, in the case of WT mice (Fig. 1F, see +TPA bars). The fold-change of these two responses is increased in the case of *Vav2*^Onc/Onc^ animals, thus indicating that catalytically active Vav2 favors the activation of SSCs under these conditions (Fig. 1F, see +TPA bars). By contrast, we observed that *Vav2*^*–/–*^;*Vav3*^*–/–*^ show increased numbers of bulge LRCs (Fig. S1F, left panels; Fig. S1G, left panel; see +TPA bars) and reduced numbers of Ki67^+^ (Fig. S1F, middle panels; Fig. S1G, right panel; see +TPA bars) cells when compared to controls. Further supporting this finding, we found using EdU labeling experiments that the CD34^+^ Itgα6^+^ cells from *Vav2*^*–/–*^;*Vav3*^*–/–*^ mice display impaired proliferation upon TPA stimulation (Fig. S1H, left panel). This defect is not due to an exhaustion of the SSC population due to asymmetric divisions as the total number of hair follicle bulge cells is not altered by the TPA treatment (Fig. S1H, right panel). Collectively, these data indicate that *Vav2*^Onc/Onc^ and *Vav2*^–/–^;*Vav3*^–/–^ SSCs exhibit opposite activation patterns upon stimulation with TPA in vivo (Table S1).

### Vav proteins regulate epidermal stem cell-dependent responses in the skin

**Fig. 2:**
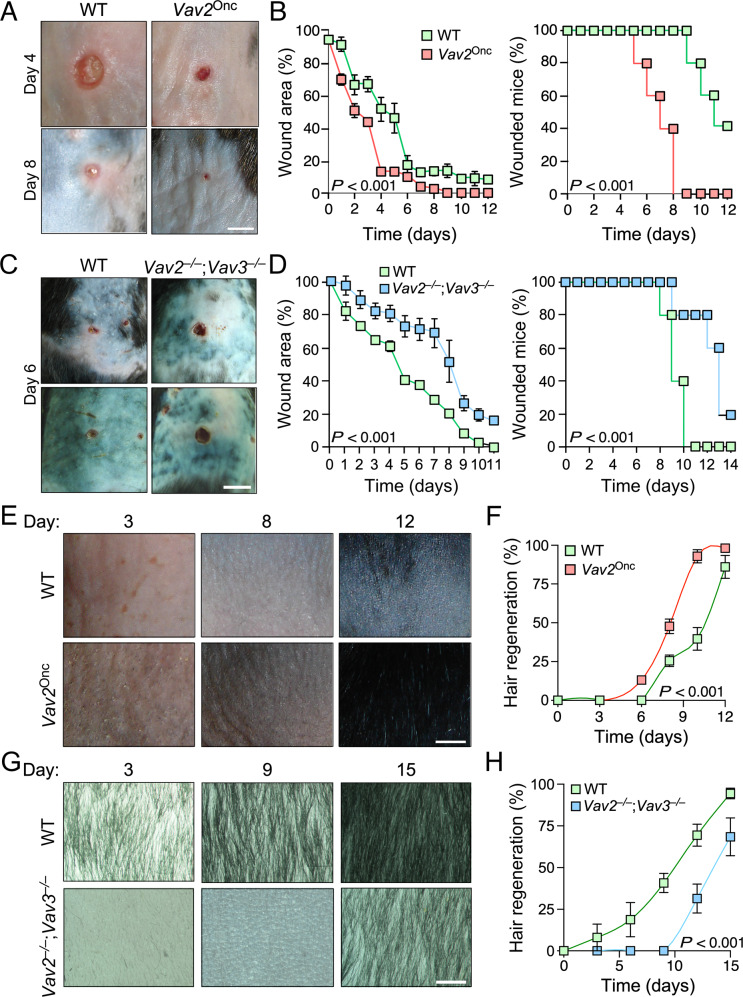
Vav proteins regulate skin stem cell-dependent responses. **A** Representative images of the dorsal skin from mice of indicated genotypes after being subjected to a wound healing experiment. Scale bar, 3 mm (*n* = 5). **B** Plots showing the wound size (left) and prevalence (right) in mice of indicated genotypes according to the data obtained in **A**. *P* < 0.001 (two-way ANOVA and Sidak’s multiple comparisons test (left), Chi-squared test (right), *n* as in **A**). **C** Representative images of the dorsal skin from mice of indicated genotypes 6 days after being subjected to wound healing experiments. Scale bar, 4 mm (*n* = 5). **D** Plots showing the wound size (left) and prevalence (right) in mice of indicated genotypes according to data obtained in **C**. *P* < 0.001 (two-way ANOVA and Sidak’s multiple comparisons test (left), Chi-squared test (right), *n* as in **C**). **E**, **G** Representative images of the hair regrowth in depilated mice of indicated genotypes. Scale bar, 2 mm. **F**, **H** Quantitation of hair regeneration kinetics obtained in **E** (**F**) and **G** (**H**). *P* < 0.001 (two-way ANOVA and Sidak’s multiple comparisons test); *n* = 6. In **B**, **D**, **F** and **H**, data represent mean ± SEM.

We next investigated whether *Vav2*^Onc/Onc^ and *Vav2*^*–/–*^;*Vav3*^*–/–*^ animals exhibited alterations in the response of the skin to excisional wound healing and hair regeneration experiments. These two challenges are heavily dependent on both the activation and mobilization of SSCs [35–38]. We observed that 8 to 10-week-old *Vav2*^Onc/Onc^ animals display accelerated healing rates when compared with their controls in response to excisional wounds in the back skin (Fig. 2A, B). Conversely, aged-matched *Vav2*^*–/–*^;*Vav3*^*–/–*^ mice show a more delayed healing when compared to their controls (Fig. 2C, D). We also evaluated SSC activation through depilation-induced hair follicle cycling, a condition that triggers the transition from the quiescence to the active state of bulge stem cells to regenerate the lost hair. In good agreement with the foregoing assays, we observed that depilated *Vav2*^Onc/Onc^ and *Vav2*^*–/–*^;*Vav3*^*–/–*^ animals could regenerate their fur faster and more slowly than the WT counterparts, respectively (Fig. 2E–H). Interestingly, these experiments revealed that hair regeneration rates are heavily dependent on the genetic background of the mouse models used, as the control animals for the knockout mouse-based experiments (C57BL/6 J background) showed faster regeneration than the controls used for the gain-of-function experiments (C57BL/6 J background) (compare Fig. 2E and G). Collectively, these data show that *Vav2*^*Onc*^ and *Vav2*^*–/–*^;*Vav3*^*–/–*^ mice display mirror-image phenotypes in terms of SSC activity and responsiveness to activation stimuli (Table S1). It is important to note, however, that these skin regeneration assays can be also influenced by factors extrinsic to SSCs (e.g., alterations in the function of mature keratinocytes or fibroblasts).

### The Vav-dependent skin phenotype is mostly keratinocyte-autonomous

**Fig. 3:**
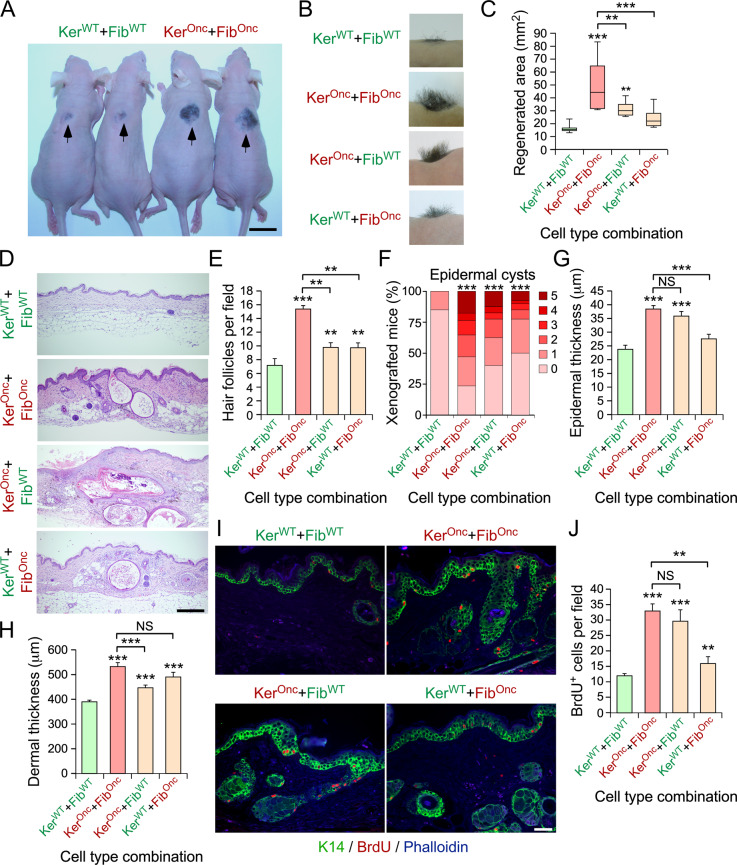
The Vav-dependent skin phenotype is mostly keratinocyte-autonomous. **A** Representative image of immunodeficient mice at the endpoint of the skin xenograft protocol performed with the indicated cell combinations. The regenerated skin area is indicated with an arrow. Scale bar, 2 cm (*n* = 5). Ker, keratinocytes; Fib, fibroblasts. **B** Representative images of hair regeneration in immunodeficient mice at the endpoint of the skin xenograft protocol shown in **A**. *n* = 5 per cell mix. **C** Quantification of the skin area regenerated by the indicated cell combinations according to the data obtained in **A**. **P < 0.01; ***P < 0.001 (ANOVA and Tukey’s HSD tests, *n* = 5). Statistical significance asterisks refer to comparisons against the Ker^WT^ + Fib^WT^ condition or against the indicated experimental condition (in brackets). **D** Representative histological sections of the skin area regenerated by the indicated transplanted cell combinations at the endpoint of the xenograft protocol. Scale bar, 100 μm (*n* = 5). **E**–**H** Quantification of hair follicle number (**E**), epidermal inclusion cysts (**F**), epidermal thickness (**G**) and dermal thickness (**H**) in the histological sections from the experiment shown in **D**. **P < 0.01; ***P < 0.001 (**E**–**G** ANOVA and Tukey’s HSD tests, **H** Chi-squared test, *n* = 5). Statistical significance asterisks refer to comparisons against the Ker^WT^ + Fib^WT^ condition or against the indicated experimental condition (in brackets). **I** Representative immunofluorescence images showing BrdU labeling (red color) and keratin 14 expression (green color) in the skin regenerated by the indicated cell combinations at the endpoint of the xenograft protocol. Phalloidin (blue color) was used for counterstaining. Scale bar, 100 μm (*n* = 5). **J** Quantification of BrdU^+^ cells the skin area regenerated by the indicated cell mixes according to data obtained in **I**. **P < 0.01; ***P < 0.001 (ANOVA and Tukey’s HSD tests, *n* = 5). Statistical significance asterisks refer to comparisons against the Ker^WT^ + Fib^WT^ condition or against the indicated experimental condition (in brackets). In **C**, **E**–**H** and **J**, data represent mean ± SEM. NS, not significant.

A feature of SSCs it the ability to regenerate the epithelial cells and hair follicles in the skin [3]. We used that feature to carry out skin xenograft experiments into immunodeficient nude mice to evaluate the skin regenerative activity of SSCs purified from *Vav2*^Onc/Onc^, *Vav2*^–/–^;*Vav3*^–/–^, and WT mice. To be effective, however, these experiments require the implantation of mixtures composed of keratinocytes (which contain the regenerative SSC pool) and fibroblasts from the donor animals. Using this approach, we found that the implantation of mixed populations of *Vav2*^Onc/Onc^ keratinocytes and fibroblasts promotes the formation of larger epidermal areas (Fig. 3A) with higher fur density (Fig. 3B) than control mixtures of WT cells in the xenografted mice (see quantitation in Fig. 3C). Histological analyses revealed that this phenotype is linked to the generation of the expected epithelial and dermal layers (Fig. 3D) and higher numbers of *de novo* hair follicle morphogenesis (Fig. 3D, E). However, we also detected the frequent formation of epidermal inclusion cysts (Fig. 3D, F), epidermal hyperplasia (Fig. 3D, G), and dermal dysplasia (Fig. 3D, H) in the samples generated by the implanted *Vav2*^Onc/Onc^ cells. Consistent with this, we also found increased levels of proliferation in the basal epithelia of those samples (Fig. 3I, J). These epidermal and dermal alterations have been previously observed in *Vav2*^Onc/Onc^ mice [23] and, probably, are caused by the deregulated Vav2^Onc^ signaling in the keratinocytes that have been generated upon the differentiation of the implanted SSCs.

The use of cell mixtures composed of *Vav2*^Onc/Onc^ keratinocytes and WT fibroblasts also leads to a statistically significant enlargement of the regenerated area of the skin (Fig. 3B, C), increased numbers of hair follicles (Fig. 3B, E), and epidermal cyst formation (Fig. 3F). However, this phenotype is milder than that found when using mixtures of *Vav2*^Onc/Onc^ cells (Fig. 3C, E and F). This indicates that this phenotype, although mostly SSC autonomous, also depends on minor contributions from the activated Vav2 pathway in fibroblasts. By contrast, we found that the epithelial hyperplasia obtained under these conditions is like that found with mixtures of *Vav2*^Onc/Onc^ cells (Fig. 3G). These results further indicate that these features of the regenerated skin are probably caused by the activity of Vav2^Onc^ in the keratinocytes generated by the implanted SSC pool. The dermal dysplasia observed under these conditions (Fig. 3H) is probably mediated by paracrine mechanisms of Vav2^Onc^-expressing keratinocytes on WT fibroblasts, as inferred from the previous observations made in the total skin and isolated keratinocytes from *Vav2*^–/–^;*Vav3*^–/–^ mice [21]. We found similar readouts when using combinations of *Vav2*^Onc/Onc^ keratinocytes and *Vav2*^–/–^;*Vav3*^–/–^ fibroblasts in these experiments (Fig. S2A–H), indicating that the dermal dysplasia found in these conditions does not require the engagement of Vav-dependent pathways in the transplanted fibroblasts. The implantation of mixtures of WT keratinocytes and *Vav2*^Onc/Onc^ fibroblasts regenerates skin areas like those observed in mixtures of WT cells (Fig. 3B, C), although we did observe a small increase in the number of hair follicles (Fig. 3E) and epidermal cysts (Fig. 3F) under these experimental conditions. In this case, we could detect the dermal dysplasia (Fig. 3H) but not epithelial hyperplasia (Fig. 3G) in the regenerated skin of the recipient animals. These results demonstrate that the effect of *Vav2*^Onc^ in the skin regenerative activity of SSCs is mostly keratinocyte autonomous, although a minor contribution of Vav2^Onc^ fibroblasts is also probably required. In addition, they indicate that the dermal dysplasia can be triggered by the constitutive activation of Vav2 catalytic activity both in keratinocytes and fibroblasts.

Finally, we performed skin xenografts using mixtures of keratinocytes and fibroblasts from *Vav2*^*–/–*^;*Vav3*^*–/–*^ mice. We did not find differences in any of the parameters analyzed when compared to WT counterparts (Fig. S2I–P). This is in agreement with previous studies indicating that the combined elimination of Vav2 and Vav3 does not elicit any overt epidermal or skin defects in mice [21]. In addition, they suggest that the reduced numbers of SSCs found in *Vav2*^*–/–*^;*Vav3*^*–/–*^ mice are still sufficient to induce normal levels of skin regeneration.

### Vav proteins regulate transcriptional programs involved in stem cell homeostasis

**Fig. 4:**
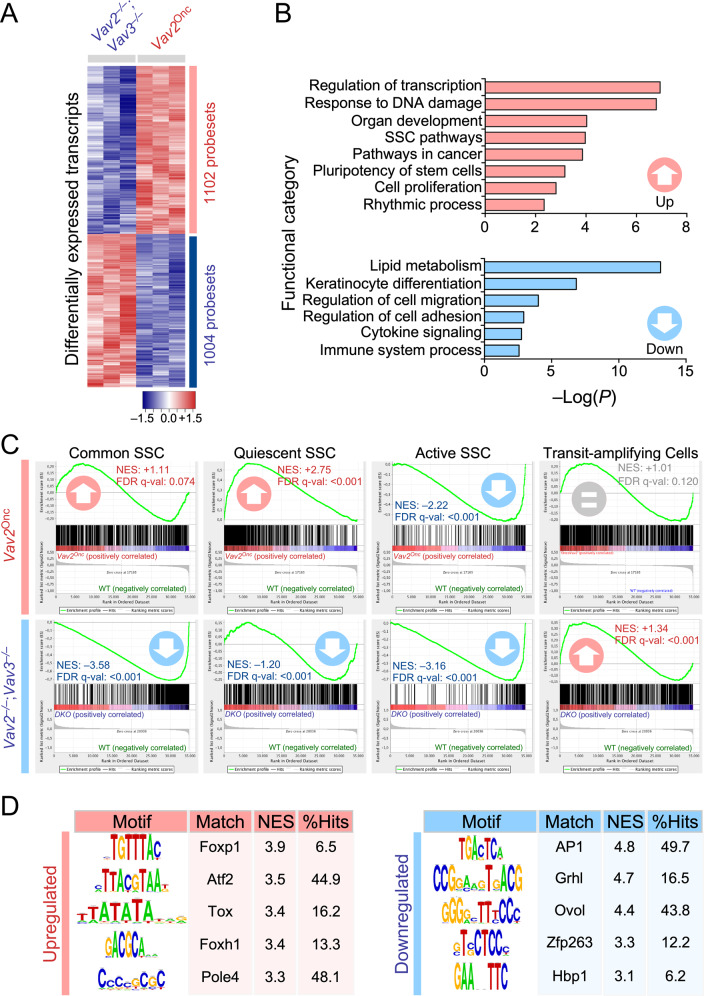
Vav proteins regulate programs involved in SSC homeostasis. **A** Heatmap showing the transcripts differentially expressed between *Vav2*^Onc/Onc^ and *Vav2*^*–/–*^;*Vav3*^*–/–*^ SSCs. The number of probe sets for the up- and downregulated fractions is indicated. **B** Main upregulated (red) and downregulated (blue) functional categories encoded by the transcriptome of *Vav2*^Onc/Onc^ SSCs. For all of them, *P* < 0.01 (Fisher’s exact test). **C** GSEA of indicated gene sets in the *Vav2*^Onc/Onc^ and *Vav2*^*–/–*^;*Vav3*^*–/–*^ SSC transcriptome. The normalized enrichment scores (NES) and false discovery rate *q*–values (FDR *q*–val) are indicated within each graph. Positive and negative enrichments are indicated with upward- and downward-pointing arrows, respectively. **D** Main enriched transcription factor binding sites in the promoters of the differentially expressed genes identified in **A**. The normalized enrichment scores (NES) are indicated. For all of them, *P* < 0.05 (iRegulon Wilcoxon rank-sum paired test).

To further shed light on the role of Vav proteins in SSCs, we performed microarray studies using RNA from CD34^+^ Itgα6^+^ epidermal cells isolated from 10-week-old *Vav2*^Onc/Onc^, *Vav2*^*–/–*^;*Vav3*^*–/–*^ SSCs, and the appropriate control mice. This age was selected because: (i) it is the time frame in which the number of SSCs differs widely between *Vav2*^Onc/Onc^ (see above, Fig. 1B) and *Vav2*^*–/–*^;*Vav3*^*–/–*^ (see above, Fig. 1D) mice. (ii) It is the in which the hair follicles are in telogen (resting phase) [38]. Given that *Vav2*^Onc/Onc^ and *Vav2*^*–/–*^;*Vav3*^*–/–*^ mice had distinct genetic backgrounds, the microarray and subsequent in silico analyses were performed in the following sequence. Firstly, we established the differentially expressed genes of the SSCs of each interrogated mouse strain with their respective controls to avoid the detection of changes exclusively due to background-related issues. Subsequently, we selected the differentially expressed genes that showed opposite patterns of expression in the SSCs from *Vav2*^Onc/Onc^ and *Vav2*^*–/–*^;*Vav3*^*–/–*^ mice. Using this approach, we found a large subset of genes (2 106 probe sets) that fulfilled these criteria (Fig. 4A and Table S2). Standard gene ontology analyses indicated that the Vav-dependent SSC upregulated transcriptional signature is highly enriched in genes associated with pluripotency, the control of transcription, DNA metabolism, and cell proliferation (Fig. 4B, top panel). By contrast, the downregulated genes are mainly associated with lipid metabolism, keratinocyte differentiation, cell adhesion and migration, and cytokine signaling (Fig. 4B, bottom panel).

To further characterize the Vav-dependent transcriptome of SSCs, we next performed gene set enrichment analyses (GSEA) to investigate the potential regulation of gene expression signatures linked to the identity, quiescence, activation and/or differentiation of SSCs [39]. We found using this in silico approach that the expression of Vav2^Onc^ promotes the upregulation of gene signatures linked to both SSC identity and quiescence (Fig. 4C, two top left panels). By contrast, it represses the expression of gene signatures associated with SSC activation (Fig. 4C, third top panel from left). The hyperactivation of Vav2 does not regulate, however, gene expression programs involved in the differentiation of SSCs to the transit-amplifying cell (TAC) state (Fig. 4C, right top panel). Conversely, the elimination of Vav2 and Vav3 leads to the downmodulation of those transcriptional signatures (Fig. 4C, two bottom left panels). However, unlike the case of *Vav2*^Onc/Onc^ SSCs, the elimination of these two GEFs promotes the upregulation of the TAC-related differentiation signature in SSCs (Fig. 4C, right bottom panel). Taken together, these analyses suggest that the catalytic activity of Vav proteins contributes to maintain the SSCs in a resting and fully undifferentiated state that, however, can rapidly be shifted into a fully activated state when challenged with external stimuli (see Figs. 1 and 2). In line with this, network analyses showed that the expression of Vav2^Onc^ drives the expression of genes involved in signaling and biological programs closely linked to SSC identity such as those encoding a variety of receptors of the Wnt pathway (Fzd3, Fzd4, Fzd5, Fzd7, Fzd8) (Fig. S3, dark green boxes) as well as transcriptional factors working at the distal downstream end of the Hippo/Yap (Tead1, Tead2, Tead4, Tcf7l2) (Fig. S3, light blue boxes), Shh (Gli1, Gli2, Gli3) (Fig. S3, light red boxes), and cAMP (Jun, Fos, Fosb, Sox9) (Fig. S3, light red boxes) pathways. They also include genes encoding protein tyrosine kinase-regulated pathways (EGFR, IGF1R, Jak2, Pik3r1) (Fig. S3, light brown boxes) and a large collection of proteins involved in the interaction with the extracellular matrix (Fig. S3, light green boxes).

We further used in silico analyses to identify the enrichment of transcription factor binding sites that could be responsible for the modulation of the Vav-dependent transcriptome in SSCs. In agreement with our GSEA analyses, we found that the upregulated genes in *Vav2*^Onc/Onc^ SSCs are enriched in binding sites for Foxp1, an essential factor for SSC quiescence [40]; the AP1-related ATF2, which regulates epidermal proliferation [41]; cell fate regulators of the Tox family [42, 43]; the pluripotency driver Foxh1[44]; and Polε4, which is a classic marker found in bulge SSC molecular signatures [45] (Fig. 4D, left panel). Conversely, the downregulated genes are enriched in binding sites for transcription factors directly involved in epidermal differentiation, including the p63 target Hbp1 [46] and members of the AP1, Grhl and Ovol families [41, 47] (Fig. 4D, right panel).

The comparison of the transcriptomes found in Vav2^Onc^-expressing SSCs (this paper) and Vav2^Onc^-expressing skin keratinocytes [23] shows limited overlap within the upregulated subset, as they only share the upregulation of 34 genes that mainly encode chromatin remodeling-factors (Fig. S4A). The downregulated subsets are also different in those two transcriptomes, although we could find in this case a higher overlap in a subset of 253 genes that mainly encode proteins associated with keratinocyte differentiation and lipid metabolism (Fig. S4B). The Vav2^Onc^-driven SSC transcriptome exhibits even less similarity with the Vav2;Vav3-regulated transcriptome previously described in the mouse 4T1 breast cancer cell line [48] (Fig. S4A, B). These results indicate that the transcriptomal changes elicited by Vav2^Onc^ are highly cell type specific. The differential regulation of representative genes detected in the Vav2^Onc^-driven SSC transcriptome (*Atf3*, *Fos*, *Fosb*, *Fzd4*, *Gli2*, *Jun;* Table S2 and Fig. S3) was corroborated by quantitative reverse transcription polymerase chain reactions (qRT-PCR) using RNA samples from Vav2^Onc^ and Vav2^WT^ SSCs (Fig. S4C).

### Vav2^Onc^ modulates the function of skin cancer stem cells

**Fig. 5:**
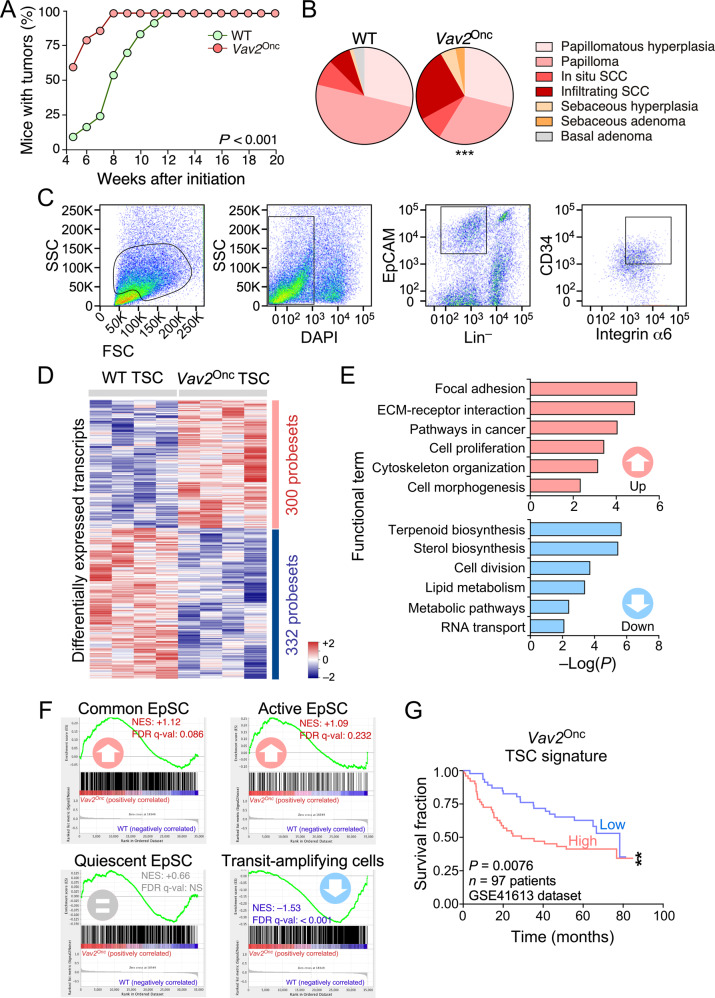
Impact of Vav2^Onc^ catalysis-dependent signaling in tumor stem cells. **A** Penetrance of tumors in DMBA-treated mice of indicated genotypes. *P* < 0.001 (Chi–squared test, *n* = 13 and 15 WT and *Vav2*^Onc/Onc^ mice, respectively). **B** Tumor type distribution of tumors at the endpoint of the experiment shown in **A**. *P* < 0.001 (Chi–squared test, *n* = 80 and 97 WT and *Vav2*^Onc/Onc^ tumors, respectively). **C** Flow cytometry gating strategy used for the isolation of tumor stem cells. **D** Heatmap showing the transcripts that are differentially expressed between *Vav2*^Onc/Onc^ and WT TSCs. The number of probe sets for the upregulated and downregulated fractions is indicated. **E** Main upregulated (red) and downregulated (blue) functional categories encoded by the transcriptome of *Vav2*^Onc/Onc^ TSCs. For all of them, *P* < 0.01 (Fisher’s exact test). **F** GSEA of indicated gene sets in the *Vav2*^Onc/Onc^ TSC transcriptome. The normalized enrichment scores (NES) and false discovery rate *q*–values (FDR *q*–val) are indicated within each graph. Positive and negative enrichments are indicated with upward and downward pointing arrows, respectively. **G** Survival plot of hnSCC patients (*n* = 97) according to the expression of the Vav2^Onc^-driven TSC transcriptional signature. The Mantel–Cox test *P* value is indicated.

Since SSCs represent one of the main cellular origins of squamous cell carcinomas [49], we next investigated the impact of endogenous Vav2^Onc^ on the biological programs of tumor stem cells (TSCs). To obtain those cells, we topically administered 7,12-dimethylbenz[a]anthracene (DMBA) to 2-month-old mice of the indicated genotypes to generate skin tumors and, subsequently, isolate TSCs from those tumors by cell sorting. We could not use in these experiments TSCs lacking Vav2 and Vav3 given the low numbers of tumors found in DMBA-treated *Vav2*^–/–^;*Vav3*^–/–^ mice [21]. In line with previous studies on newborn mice [23], we found that DMBA-treated *Vav2*^Onc/Onc^ animals develop skin tumors with faster kinetics (Fig. 5A) and higher malignancy (Fig. 5B) than controls. Consistent with this, we observed an enlargement of the percentage of tumors that have acquired the infiltrating squamous cell carcinoma status in the case of *Vav2*^Onc/Onc^ mice (Fig. 5B). Despite this, we did not detect any statistically significant difference in the total number and size of the tumors obtained in each genotype (Fig. S5A, B). This suggests that Vav2^Onc^ favors the initiation rather than the promotion of this type of tumors. To isolate TSCs, we collected the tumors from *Vav2*^Onc/Onc^ and WT mice and, after homogenization, sorted the CD31^–^ CD45^–^ CD140a^–^ EpCAM^+^ CD34^+^ Itgα6^+^ cell fraction by flow cytometry (Fig. 5C). We obtained similar numbers of TSCs regardless of the genotype of those mice (data not shown, *n* = 6 per genotype). Genome-wide expression analyses of this purified TSC population revealed major transcriptomic differences between those two TSC populations and normal, WT SSCs (Fig. S5C and Table S3). As expected, the TSC-specific transcriptome encompasses a large collection of genes involved in functions typically associated with tumorigenic processes such as cell division, cytoskeletal organization, RNA metabolism, protein translation, cell signaling, and metabolic rewiring (Table S3). However, further analyses indicated that the expression of Vav2^Onc^ leads to the specific deregulation of a limited number of genes (632 probe sets) in TSCs when compared to those present in tumors generated in WT mice (Fig. 5D and Table S4). Functional enrichment analyses revealed that this Vav2^Onc^-specific transcriptomal subset includes upregulated genes linked to cytoskeletal-related processes (focal adhesion, interaction of cells with the extracellular matrix, cytoskeletal organization), cell proliferation, and cancer-related pathways (Fig. 5E, top panel). It also includes downregulated genes mostly associated with specific metabolic pathways (terpenoid and sterol biosynthesis, lipid metabolism) and RNA transport (Fig. 5E, bottom panel). Interestingly, GSEA analyses revealed that the fraction of the TSC transcriptome that is specifically deregulated by Vav2^Onc^ is also enriched in gene signatures associated with the determination of the stem cell identity that were previously observed in the Vav-dependent transcriptome of normal SSCs (Fig. 5F, top left panel). However, unlike this latter case, we found that the genes signatures linked to the activated (Fig. 5F, top right panel) and quiescent state (Fig. 5F, bottom left panel) of SSCs are enriched and not enriched in Vav2^Onc^-expressing TSCs, respectively. These cells also show the repression of the gene signature associated with the transit-amplifying state (Fig. 5F, bottom right panel) which is not detected in *Vav2*^Onc/Onc^ SSCs and that is upregulated in *Vav2*^–/–^;*Vav3*^–/–^ SSCs (Fig. 4C). In line with this, we have found a very limited overlap of the Vav2^Onc^-driven transcriptome of TSCs with those previously described in SSCs, adult keratinocytes, and 4T1 cells (Figs. S4A, B and S5D). This further suggests that the transcriptomal changes induced by Vav proteins are highly dependent on the cell background context.

Given that the presence of signatures associated to stemness and reduced differentiation are usually associated with poor prognosis in squamous cell carcinoma patients [50], we finally evaluated whether the transcriptional program elicited by Vav2^Onc^ in TSCs could stratify patients according to long-term survival. As mortality rates in cutaneous SCC patients are too low to perform meaningful survival analyses [51], we tested the Vav2^Onc^–TSC signature in head-and-neck squamous cell carcinoma patients. We have shown before that Vav2 plays pro-tumorigenic roles in this tumor using pathways like those found in skin keratinocytes [23, 52]. These analyses indicate that high levels of the Vav2^Onc^–TSC signature correlate with shorter patient survival (Fig. 5G), thus proving the prognostic relevance of the transcriptional program identified here.

## Discussion

In this work, we have shown that SSC homeostasis relies on the signal transduction mediated by the Rho exchange factors Vav2 and Vav3. Through flow cytometry-, immunofluorescence- and in vitro cell culture-based experiments, we have demonstrated that the upregulation of the catalytic activity of Vav2 favors both the expansion and the subsequent stabilization of the enlarged SSC population in mice. Importantly, our microarray analyses indicate that these features are associated with the upregulation of transcripts involved in both SSC identity and quiescence. Despite this, Vav2^Onc^-expressing SSCs are also associated with a more responsive state to external signals, as demonstrated by our TPA-stimulation, wound healing, hair regeneration, and skin reconstitution experiments. Conversely, the loss of both Vav2 and Vav3 results in the depletion of transcripts linked to SSC homeostasis and the upregulation of genes associated with SSC differentiation. The loss of Vav proteins is also associated with a lower responsiveness of SSCs to TPA and regenerative stimuli such as wound healing and hair regeneration. However, and in agreement with the lack of dysfunctions in the skin of *Vav2*^–/–^;*Vav3*^–/–^ mice [21], we have not seen any defect in the skin reconstitution of the skin in our transplantation experiments. This indicates that, despite the low numbers (see above, Fig. S1D), the SSCs present in these animals maintain the ability to regenerate the skin under normal physiological conditions. However, the Vav-deficient SSC do show defects under more extreme regenerative challenges such as those imposed by wound-healing and hair regrowth. The comparison of the fraction of the SSC transcriptome that shows opposite behavior in the presence of Vav2^Onc^ and in the absence of Vav proteins indicates that the effect of the dysregulation of Vav activity in these cells is associated with the regulation of bona-fide transcriptional regulators involved in skin stem cell pluripotency. Collectively, these results indicate that the activity of Vav proteins is important to: (i) Generate proper numbers of SSCs during the postnatal expansion phase. (ii) Maintain the SSC pool in a pluripotent, ready-to-go state. (iii) Ensure proper responses to external challenges such as those imposed by wound healing and hair regrowth upon skin depilation. Importantly, we have seen that the effect of the loss of function of the Vav family is only apparent when both Vav2 and Vav3 are missing in mice. These data indicate that these two related GEFs probably work redundantly in the context of SSCs.

Our transplantation experiments with mixed populations of keratinocytes (which include the SSC fraction responsible for skin regeneration) and fibroblasts have also helped us to further dissect the specific contribution of those cell populations to skin formation. The data obtained indicate that the expression of Vav2^Onc^ in the SSC/keratinocyte pool is self-sufficient to reproduce the skin phenotype found upon the concurrent transplantation of both *Vav2*^Onc/Onc^ keratinocytes and *Vav2*^Onc/Onc^ fibroblasts. However, it is worth noting that the transplantation of *Vav2*^Onc/Onc^ fibroblasts also leads to a minor increase in the number of hair follicles that are formed in the regenerated skin when compared to WT controls. By contrast, they do not favor any statistically significant expansion of the total area of the skin that is regenerated in these experiments. The normal regenerative activity of the skin found in our xenotransplants experiments when using *Vav2*^–/–^;*Vav3*^–/–^ cells also suggest that, in this case, the function of the wild-type Vav proteins can be compensated by additional Rho GEFs expressed in SSCs. Interestingly, the analysis of the cell progeny generated by these skin regeneration studies provides further light on the cell types responsible for the skin phenotype previously observed in *Vav2*^Onc/Onc^ mice [23]. Thus, consistent with our previous results [23], the data obtained with the xenotransplants experiments clearly indicate that the epidermal hyperplasia and dermal dysplasia observed in those animals is dependent on the abnormal signaling triggered by *Vav2*^Onc^ in keratinocytes. The effect of *Vav2*^Onc/Onc^ keratinocytes on fibroblasts is probably mediated by the Vav-dependent autocrine and paracrine loops present in the former cells that have been previously described in an earlier study [21]. These data indicate that the response of fibroblasts to the signals delivered by Vav2^Onc^ keratinocytes can take place in the absence of endogenous Vav2 and Vav3. However, our present results also suggest that the *Vav2*^Onc^ signaling in fibroblasts can promote per se dermal dysplasia without any noticeable effects on the thickness of the epithelial layer. Our transplantation results with *Vav2*^–/–^;*Vav3*^–/–^ cells have also revealed that, as inferred from the analysis of the skin of *Vav2*^–/–^;*Vav3*^–/–^ knockout mice [21], the function of the endogenous versions of these two Vav family members is not required to maintain skin homeostasis under normal conditions. These observations further suggest that there must be additional Rho GEFs that will cooperate with Vav proteins in the maintenance of steady-state conditions of this tissue.

The present study has also revealed that the hyperactivation of the catalytic activity of Vav2 does not lead to significant increases in the number of TSCs, at least in the context of the carcinogen-induced tumors utilized in this work. Despite this, we observed that the expression of Vav2^Onc^ drives specific transcriptomal changes in TSCs. Although most of them are TSC-specific (see below), it is worth noting that those changes include the enrichment of gene signatures linked to the determination of the identity of stem cells previously observed in Vav2^Onc^-expressing normal SSCs. This suggests that Vav2 signaling has probably relevant roles in both the establishment and maintenance of this biological program (Fig. 5F, top panels). Despite this, the transcriptomal program of *Vav2*^Onc/Onc^ TSCs and SSCs is quite different when we consider other SSC-linked biological programs such as those associated with quiescence, activation, and transit-amplifying states.

In line with the foregoing observations, our results indicate that Vav2^Onc^ engages quite different, cell type-specific biological programs in the epidermis. Thus, in the case of keratinocytes, we have demonstrated that the expression of Vav2^Onc^ promotes a regenerative proliferation state characterized by high levels of keratinocyte proliferation and undifferentiation. These cells also acquire stem cell- and TSC-like features according to previous genome-wide transcriptomal analyses [23]. These programs are nucleated by several transcriptional factors such as Myc, YAP/TAZ, AP1 family members, and E2F [23]. By contrast, we have observed that Vav2^Onc^-expressing SSCs do not acquire the features TSC-like features seen in keratinocytes. On the contrary, they acquire features mostly associated with cytoskeletal change, an active proliferative state, and the inhibition of gene programs linked to transit-amplifying cells. The comparison of the Vav family transcriptomes found in the skin, SSCs, and other tumor types also indicate that these GEFs engage cell- and tumor-type-specific biological programs. Collectively, these results indicate that the Vav genetic programs activated in the skin are probably highly dependent on the cell background context. They also suggest that we could probably hit skin tumors pharmacologically using combinations of drugs targeting different Vav-dependent Achilles’ heels specific for TSCs, transformed keratinocytes and, possibly, fibroblasts.

It is difficult to foresee at present the signaling pathways involved in the engagement of the Vav-dependent biological programs in SSCs. However, the mirror-image effects found in most of the experiments using gain- and loss-of-function models suggest that the action of Vav2 must be heavily dependent on the stimulation of catalysis-dependent pathways. In line with this, we have previously shown that the positive action of endogenous Vav2^Onc^ and Vav2^WT^ in skin tumorigenesis is also catalysis-dependent [21, 23, 30]. Consistent with this hypothesis, we have previously described that the swapping of Vav2 by a catalytically impaired mutant version of Vav2 (with the L332A point mutation in the catalytic Dbl-homology domain) that keeps all the adapter domains intact blocks skin tumorigenesis at levels comparable to the total depletion of both Vav2 and Vav3 [23]. In terms of the potential GTPase substrates involved, we have also shown that endogenous Vav2^Onc^ triggers the stimulation of Rac1 and, to a lower extent RhoA in primary keratinocytes [23]. By contrast, it does not have any statistically significant effect on Cdc42 in the same cell system [23]. In agreement with these observations, we have demonstrated using organotypic 3D models that the epithelial hyperplasia induced by Vav2^Onc^-expressing primary keratinocytes is Rac1–Pak1-dependent and, to a lesser extent, RhoA–Rock-dependent [23]. Likewise, we have shown that the signaling and proliferative defects found in primary *Vav2*^–/–^;*Vav3*^–/–^ keratinocytes are connected to defective Rac1 activation [21]. All these data suggest that the roles of Vav2 and Vav3 proteins in SSCs must be catalytic-dependent and, most likely, Rac1-dependent. We cannot formally rule out, however, that the adapter-like function of these proteins could make a marginal role in this process. It is also worth noting that we have focused our analyses on transcriptomal programs, given the difficulty in analyzing other biological parameters for single SSCs in the right tissue context. Due to this, we cannot exclude the possibility that some of the readouts analyzed could be influenced by non-transcriptional mediated mechanisms such as, for example, the well-known role of Rho GTPases in cytoskeletal organization. These pathways can indeed affect some of the biological readouts used in this study such as the mobilization of SSCs upon the challenges imposed by the TPA stimulation or skin regeneration processes. However, it is important to notice that the Vav-dependent transcriptome of SSCs does not show any enrichment in genes under the control of F–actin-regulated transcriptional regulators such as the serum response factor (Fig. 4D). Furthermore, genes associated with cell migration and adhesion are specifically concentrated in the downregulated subset of the Vav-dependent SSC transcriptome (Fig. 4B). By contrast, the Vav2^Onc^-driven transcriptome of TSCs does show a significant enrichment in genes linked to focal adhesion dynamics, extracellular matrix interactions, and cytoskeletal organization (Fig. 5E). It is possible to hypothesize, therefore, that the need of cytoskeletal-related functions will be quite different in normal and tumoral skin stem cells. Further studies will be required to identify all the spectrum of proximal and distal Rac1 effectors involved in this process.

## Materials and methods

### Ethics statement

All mouse experiments were performed according to protocols approved by the Bioethics Committee of the University of Salamanca and the animal experimentation authorities of the autonomous government of Castilla y León (Spain). We have not utilized patients or patient-derived samples in this work.

### Mouse strains

*Vav2*^–/–^ (C57BL/10 J background), *Vav3*^–/–^ (C57BL/10 J background), *Vav2*^–/–^;*Vav3*^–/–^ (C57BL/10 J background), and Vav2^Onc^ (C57BL/6 J background) male mice have been previously described [21, 23, 24]. In all cases, the animals were kept in ventilated rooms in a pathogen-free facility under controlled temperature (23 °C), humidity (50%), and illumination (12-hour-light/12-hour-dark cycle) conditions.

### Animal studies

For label-retaining cell assays, 50 mg/kg of BrdU (ThermoFisher Scientific, Catalog No. B23151) were injected intraperitoneally in 10-day-old mice every 12 h for a total of 4 injections and chased after 80 days. For epidermal stem cell proliferation assays, 1 mg of EdU (Invitrogen, Catalog No. A10044) was injected intraperitoneally 1 h before euthanasia in mice of indicated ages. Wound healing assays were performed in 8-week-old mice. To this end, animals were shaved on the back and, two days later, used to generate an incision in the dorsal skin using a biopsy punch (Stiefel, Catalog No. BI1500). Healing was monitored daily since then using a digital caliper. For hair regeneration assays, 8-week-old mice were shaved and the regrowth of the hair was followed up daily and quantified using ImageJ2 (version 2.3.0/1.53 f). For skin stimulation experiments, the dorsal skin of shaved animals was treated with 200 μL of an acetone solution of 3.3 ×10^–5^ M TPA (Sigma, Catalog No. P8139) and collected 24 h later after euthanized the animals. For skin xenograft experiments, keratinocytes and fibroblasts were isolated from donor mice as indicated previously [21]. Briefly, the skin from newborn mice was isolated and incubated in CnT-07 medium (CELLnTEC, Catalog No. CnT-07) with 5 mg/mL of dispase (Roche, Catalog No. 04942078001) overnight at 4 °C to separate the epidermis from the dermis. For keratinocyte isolation, the epidermis was then incubated with accutase (CELLnTEC, Catalog No. CnT-Accutase-100) for 30 min at 37 °C. For fibroblast isolation, the dermis was digested in DMEM (ThermoFisher Scientific, Catalog No. 11995065) with 0.25% collagenase (Sigma, Catalog No. C5138) for 1 h at room temperature. All the keratinocytes resulting from one newborn mouse plus 4 ×10^6^ dermal cells were combined in a cell suspension for each xenograft. 8-week-old nude mice (Charles River, Catalog No. NU/NU) were anesthetized with 100 μL of a mixture of 25 mg/mL ketamine (Merial, Catalog No. Imalgene 1000), 2 mg/mL diazepam (Roche, Catalog No. Valium) and 0.1 mg/mL atropine (Braun, Catalog No. Atropine B) and placed on a heating pad. After cleaning the dorsal surface with iodopovidone, a 1-cm long incision in the skin was made to insert a skin xenograft chamber as reported before [53]. The chamber was then secured with stitches and the cell suspension introduced. One week after the intervention, mice were anesthetized and the chamber removed. Six weeks later, skin reconstitution was considered complete and animals were injected intraperitoneally with 50 mg/kg of BrdU and sacrificed 1 h later. Xenografted skin was then taken for histological and immunohistochemical studies (see below).

Skin carcinogenesis experiments were performed as previously described [21]. Briefly, 2-month-old mice were treated biweekly with 5 μg of DMBA in 200 μL of acetone for 20 weeks. The number, size (measured with a digital caliper) and incidence of tumors was determined weekly. At the end of the experiment, animals euthanized and tumors subjected to either histological or flow cytometry analyses.

### Epidermal stem cell isolation

The isolation of epidermal stem cells was carried out as previously described [54]. Briefly, the back skin of animals was excised, cleaned, and digested in 0.25% trypsin (ThermoFisher Scientific, Catalog No. 25200056) overnight at 4 °C. This cell suspension was then filtered, resuspended in EMEM (Lonza, Catalog No. BE06-174G) with 15% fetal bovine serum (ThermoFisher Scientific, Catalog No. 10500064), incubated for 30 min on ice with biotin-conjugated anti-CD34 (1:50 dilution, eBioscience, Catalog No. 13-0341-85), and subsequently incubated for 30 min with both APC-conjugated streptavidin (1:300 dilution, BD Biosciences, Catalog No. 554067) and PE-conjugated anti-CD49f (1:200 dilution, AbD Serotec, Catalog No. MCA699PE). Finally, 4’,6-diamidino-2-phenylindole incubation (0.1 ng/mL) was added for 5 min to mark (and exclude from the final count) dead cells. Positive cells for CD34 and CD49f were isolated using a FACSAria III flow cytometer (BD Biosciences) and analyzed with the FlowJo software (BD Biosciences).

### Colony formation assays

Keratinocytes were isolated as indicated before, counted, and plated onto six-well plates at a density of 500 cells per well in CnT-07 medium. 10-14 days later, cells were fixed with 4% paraformaldehyde for 10 min and stained with Giemsa (1:10, Merck, Catalog No. 32884) for another 10 min. Plates were washed with distilled water, left to dry, and colonies counted.

### Whole mount immunohistochemical analyses

Tail skin was incubated in 5 mM ethylenediaminetetraacetic acid (EDTA) in phosphate-buffered saline solution at 37 °C for 4 h to separate de epidermis from the dermis. The epidermis was then fixed at room temperature in 4% paraformaldehyde for 2 h, permeabilized in 0.2% Triton X-100 (Sigma, Catalog No. T8787) for 15 min and blocked in 2% horse serum (ThermoFisher Scientific, Catalog No. 16050122) for 30 min using 10 min washing steps with phosphate-buffered saline solution in between. Incubation with the primary antibody was performed in 2% horse serum overnight at room temperature using the following antibodies: BrdU (1:100 dilution, BD Biosciences, Catalog No. 347580), Ki67 (1:100 dilution, Novocastra, Catalog No. NCL-L-Ki67-MM1), K14 (1:400 dilution, Covance, Catalog No. PRB-155P). Secondary antibody incubation was performed on the next day under the same conditions with Alexa Fluor 488 (1:400 dilution, Invitrogen, Catalog No. A21202) and Cy3 (1:400 dilution, Jackson Immunoresearch, Catalog No. 111-165-144). After washing, the preparations were stained with 4’,6-diamidino-2-phenylindole incubation (0.1 ng/mL) for 1 h and mounted using Mowiol (Calbiochem, Catalog No 9002–89–5). Samples were analyzed with a Leica TCS SP5 confocal microscope. Images were captured with LAS AF software (version 2.6.0.72266, Leica).

### Epidermal stem cell proliferation

DNA replication in proliferating cells was assessed using the Click-iT EdU Alexa Fluor 647 Flow Cytometry Assay Kit (ThermoFisher Scientific, Catalog No. C10424) according to manufacturer’s instructions.

### Skin histological and histochemical analyses

For histological studies, tissues were extracted, fixed in 4% paraformaldehyde, paraffin embedded, cut in 2-3 µm thick sections, and stained with hematoxylin-eosin. Images were captured using an Olympus BX51 microscope coupled to an Olympus DP70 digital camera. The quantification of the thickness of the epidermis and dermis thickness quantification was done using the ImageJ software. Immunofluorescent staining was performed using a Ventana Discovery Ultra instrument (Roche, Catalog No. 05987750001). Paraffin–embedded sections were dewaxed, subjected to Tris EDTA [pH 8.0] for heat–induced antigen retrieval, and incubated for 40 min with the appropriate primary antibody to BrdU (1:100 dilution) or K14 (1:400 dilution). The sections were then incubated in a humid chamber for 1 h with secondary antibodies to rabbit and mouse IgGs labeled with Alexa Fluor 488 (1:200 dilution, ThermoFisher Scientific, Catalog No. A21206) and Cy3 (1:200 dilution, Jackson ImmunoResearch, Catalog No. 115–165–146), respectively. After a 20-min long incubation with rhodamine-labeled phalloidin (Invitrogen; diluted 1:200 in TBS–T and 2% bovine serum albumin), sections were mounted onto microscope slides with Mowiol (Calbiochem, Catalog No. 9002–89–5). Samples were analyzed with a Leica TCS SP5 confocal microscope. Images were captured with LAS AF software (version 2.6.0.72266, Leica).

### RNA extraction and transcriptome profiling

Epidermal stem cells were lysed in RLT buffer and RNA was extracted using the QIAGEN RNeasy Micro Kit (QIAGEN, Catalog No. 74004) according to manufacturer’s instructions. Purified RNA was processed as indicated elsewhere [54] and hybridized to Affymetrix GeneChip Mouse Gene 1.0 ST microarrays. R was used to perform the bioinformatic analyses. Signal intensity values were obtained from CEL files after applying the Robust Multichip Average function from the ‘affy’ package for background adjustment, quantile normalization and summarization [55]. Differentially expressed genes were identified using linear models for microarray data (*limma*) [56], and adjusting *P* values for multiple comparisons by applying the Benjamini-Hochberg correction method [57]. To rule out the effects associated with the genetic background of the mice strains, the expression profiles were normalized using WT C57BL/6 J and C57BL/10 J mice. The *heatmap3* package [58] was used to generate the expression heatmaps for the indicated genes. Functional annotation was performed using ToppFun [59]. An FDR q-value of 0.05 was set as threshold for statistical significance. GSEA was carried out with the described gene sets using gene set permutations (*n* = 1000) for the assessment of significance and signal-to-noise metric for ranking genes [60]. The different SSC and TSC gene sets used in GSEA analyses were obtained from [39] and [61], respectively. Known functional interactions among relevant genes were obtained through the String tool [62]. Cytoscape software was used to perform network data integration and visualization [63]. For the discovery of transcription factor binding motifs in the promoters of the co-regulated genes, the iRegulon software was used [64]. A collection of 9 713 position weight matrices (PWMs) was applied to analyze 10 kb centered around the TSS. With a maximum false discovery rate (FDR) on motif similarity below 0.001, we performed motif detection, track discovery, motif-to-factor mapping and target detection. The cross-comparison among the different Vav-regulated transcriptional signatures was performed using the gene sets identified in [23] (Vav2^Onc^ skin)[48], (Vav2;Vav3 knockdown breast cancer cell), and this study (Vav2^Onc^ SSC, Vav2^Onc^ TSC). Overall survival analyses were performed through Kaplan-Meier estimates according to the enrichment level of the indicated transcriptional signature using the GSE41613 (*n* = 97 samples) head-and-neck SCC dataset. The median of the enrichment distribution for the signature was used to establish the low and high expression groups and, subsequently, the Mantel-Cox test was applied to statistically validate the differences between the survival distributions.

### Determination of mRNA expression levels

Epidermal stem cells were lysed in RLT buffer and RNA was extracted as indicated in the previous section. Reverse transcription and cDNA amplification was carried out using the QuantiTect Whole Transcriptome Kit (Qiagen, Catalog No. 207043). qRT-PCR was performed using iQ SYBR Green Supermix (Bio-Rad, Catalog No. 1708880) and the StepOnePlus Real-Time PCR System (Applied BioSystems). Raw data were analyzed using the StepOne software v2.1 (Applied Biosystems), using the abundance of the endogenous *Gapdh* mRNA as internal normalization control. Primers used for transcript quantitation included: 5’-AGG AAG AGC TGA GAT TCG CC-3’ (forward, *Atf3*), 5’-GGG GTC TGT TGA CGG TA-3’ (reverse, *Atf3*), 5′-GGC TTC CCA GAG ATG TCT-3′ (forward, *Fos*), 5’-TGC TGA TGC TCT TGA CTG GC-3’ (reverse, *Fos*), 5’-GAA CGC CTG GAG TTT GTC CT-3’ (forward, *Fosb*), 5’- TCC TTA GCG GAT GTT GAC CC-3’ (reverse, *Fosb*), 5’-TGC CAG AAC CTC GGC TAC A-3’ (forward, *Fzd4*), 5’-ATG AGC GGC GTG AAA GTT GT-3’ (reverse, *Fzd4*), 5’-TAC CTC AAC CCT GTG GAT GC-3’ (forward, *Gli2*), 5’-CTA CCA GCG AGT TGG GAG AG-3’ (reverse, *Gli2*), 5’-GCA CAT CAC CAC TAC ACC GA-3’ (forward, *Jun*), 5’-GGG AAG CGT GTT CTG GCT AT-3’ (reverse, *Jun*), 5’-CTC CTG CAC CAC CAA CTG CT-3’ (forward, *Gapdh*), 5’-GGG CCA TCC ACA GTC TTC TG-3’ (reverse, *Gapdh*).

### Isolation of tumor stem cells

At the endpoint of skin carcinogenesis experiments (see above), tumors were isolated, minced and incubated overnight at 4 °C in Hanks’ Balanced Salt Solution (HBSS) (ThermoFisher Scientific, Catalog No. 24020117) with 0.25% of collagenase I (Sigma-Aldrich, Catalog No. C9891). Next day, the leftover pieces were processed using a gentle- MACS dissociator (Miltenyi Biotec, Catalog No. 130-093-235) and gentleMACS C tubes (Myltenyi Biotec, Catalog No. 130- 093-237). The cell suspension was then filtered, resuspended in EMEM (Lonza, Catalog No. BE06-174G) with 15% fetal bovine serum (ThermoFisher Scientific, Catalog No. 10500064), incubated for 30 min on ice with biotin-conjugated anti-CD34 (1:50 dilution, eBioscience, Catalog No. 13-0341-85), and subsequently incubated for 30 min with FITC-conjugated streptavidin (1:100 dilution, BioLegend, Catalog No. 405201), PE-conjugated anti-CD49f (1:200 dilution, AbD Serotec, Catalog No. MCA699PE), APC-conjugated anti-CD31 (1:200 dilution, BD Pharmingen, Catalog No. 551262), APC-conjugated anti-CD45 (1:200 dilution, BD Pharmingen, Catalog No. 559864), APC-conjugated anti-CD140a (1:200 dilution, BD Pharmingen, Catalog No. 562777), and APC/Cy7-conjugated anti-EpCAM (1:200 dilution, BioLegend, Catalog No. 118217). Finally, 4’,6-diamidino-2-phenylindole incubation (0.1 ng/mL) was added for 5 min to mark dead cells. Positive cells for EpCAM, CD34 and CD49f, and negative for the rest of markers, were isolated using a FACSAria III flow cytometer (BD Biosciences) and analyzed with the FlowJo software (BD Biosciences). Upon isolation and RNA extraction, transcriptomal and in silico analyses were performed as indicated above.

### Statistics

The number of biological replicates (*n*), the type of statistical tests performed, and the statistical significance are indicated for each experiment either in the figure legends or in the main text. Sample size estimation was based on power calculation and performed using the daewr R package. In cases were that estimation could not be performed, we used at least *n* = 3 biological replicates. Animals were randomly allocated in experimental groups. No blinding was performed. No samples were excluded from the analyses. Data normality and equality of variances were analyzed with Shapiro-Wilk and Bartlett’s tests, respectively. Parametric distributions were analyzed using Student’s *t*-test (when comparing two experimental groups) or ANOVA followed by either Dunnett’s (when comparing more than two experimental groups with a single control group) or Tukey’s HSD test (when comparing more than two experimental groups with every other group). Sidak’s multiple comparison test was used when comparing different sets of means. The chi-squared test was used to determine the significance of the differences between expected and observed frequencies. In all cases, values were considered significant when *P* ≤ 0.05. Data obtained are given as the mean ± SEM.

## Materials availability

All relevant data are available from the corresponding author upon reasonable request. A Materials Transfer Agreement could be required in the case of potential commercial applications.

## Supplementary information

Supplementary Information

Dataset 1

Dataset 2

Dataset 3

## Data Availability

Microarray data reported in this paper have been deposited in the GEO database (https://www.ncbi.nlm.nih.gov/geo/) under the accession number GSE180946.
