# Supplementary material for: The Rho guanosine nucleotide exchange factors Vav2 and Vav3 modulate epidermal stem cell function
Source: Oncogene. 2022 May 9;41(24):3341–54. doi: 10.1038/s41388-022-02341-7 (PMC9187518; doi:10.1038/s41388-022-02341-7)
Supplement: Supplementary file 1 — Supplementary Information [file 41388_2022_2341_MOESM1_ESM.pdf]

Supplementary Information for

THE Rho GUANOSINE NUCLEOTIDE EXCHANGE FACTORS Vav2 AND Vav3 MODULATE EPIDERMAL STEM CELL FUNCTION

by

L.F. Lorenzo-Martín *et al*.

This PDF file includes (in the order in which they are mentioned in the main text):

Figures S1 to S5

Table S1

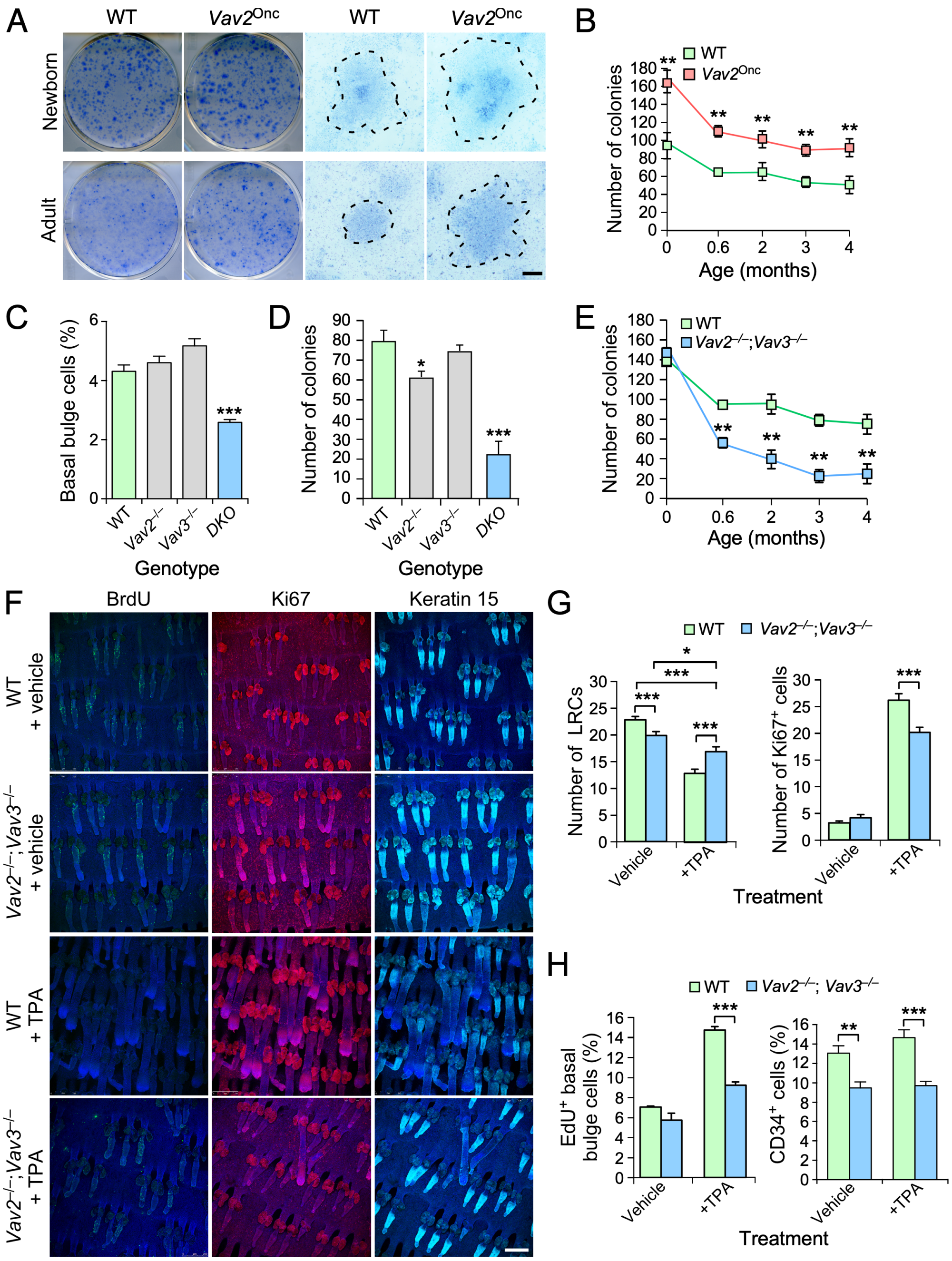

FIGURE S1. Vav function regulates SSC numbers.

(A) Representative images of the colonies (stained with Giemsa) formed by epidermal cells from mice of indicated genotypes and ages. The right panels show magnifications of representative colonies obtained in these assays (boundaries of the colony are indicated with a dashed line). Scale bar, 50 µm ($n = 4$).

(B) Quantification of the colony-forming efficiency of epidermal cells isolated from mice of indicated genotypes and ages according to the data obtained in A. **, $P < 0.01$ (two-way ANOVA and Sidak's multiple comparisons test, $n =$ 4 per genotype and time point).

(C) Quantification of the abundance of CD34$^+$ Itga6$^+$ cells in the epidermis of mice of indicated genotypes. *, $P <$ 0.05; **, $P < 0.01$; ***, $P < 0.001$ (two-way ANOVA and Sidak's multiple comparisons test, $n = 4$ per genotype and age). DKO, *Vav2*$^{-/-}$;*Vav3*$^{-/-}$ mice.

(D) Quantification of the colony-forming efficiency of epidermal cells isolated from mice of indicated genotypes. *, $P < 0.05$; ***, $P < 0.001$ (ANOVA and Dunnett's multiple comparison test, $n = 3$).

(E) Quantification of the colony-forming efficiency of epidermal cells isolated from mice of indicated genotypes and ages. **, $P < 0.01$ (two-way ANOVA and Sidak's multiple comparisons test, $n = 3$).

(F) Representative whole mount immunofluorescence images showing the presence of BrdU (green color) and the expression of Ki67 (red color) and keratin 15 (blue color) in the tail epidermis from mice of indicated genotypes. Scale bar, 200 μm ($n = 6$).

(G) Quantification of the number of label-retaining (left) and $Ki67^+$ (right) cells in the hair follicle bulge of mice of the indicated genotypes upon the indicated treatments according to the data gathered from the experiment shown in panel F. *, $P < 0.05$; ***, $P < 0.001$ (ANOVA and Tukey's HSD tests, $n = 6$).

(H) Quantification of the abundance of total $CD34^+$ (left) and $CD34^+$ $Itga6^+$ EdU^+ (right) cells in the epidermis of mice of the indicated genotypes upon the indicated treatments. **, $P < 0.01$; ***, $P < 0.001$ (Student's *t*-test, $n = 6$).

In B, C, D, E, G and H, data represent mean ± SEM.

TABLE S1. Summary of the SSC-related phenotypes found in *Vav2*$^{Onc/Onc}$ and *Vav2*$^{-/-}$;*Vav3*$^{-/-}$ mice. Upregulation and downregulation events are indicated by red and blue arrows, respectively. Grades of blue represent the relative intensity of the interrogated biological parameter. No effect is depicted as a "=" symbol. The figure number in which the experimental data are found in indicated on the right column (left, data from *Vav2*$^{Onc/Onc}$ mice; right, data from *Vav2*$^{-/-}$;*Vav3*$^{-/-}$ mice.

	Vav2$^{Onc/Onc}$	*Vav2*$^{-/-}$;*Vav3*$^{-/-}$	Figure number
Number of bulge SSCs	↑	↓	1B and 1D
EdU^{+} SSCs	↑	=	1C and S1H
Number of bulge LRCs	↑	↓	1E,F and S1F,G
Number of bulge Ki67^{+} cells	↑	=	1E,F and S1F,G
Number of bulge LRCs upon TPA stimulation	↓	↑	1E,F and S1F,G
Bulge Ki67^{+} cells upon TPA stimulation	↑	↓	1E,F and S1F,G
in vivo wound healing	↑	↓	2A,B and 2C,D
Hair regeneration	↑	↓	2E,F and 2G,H

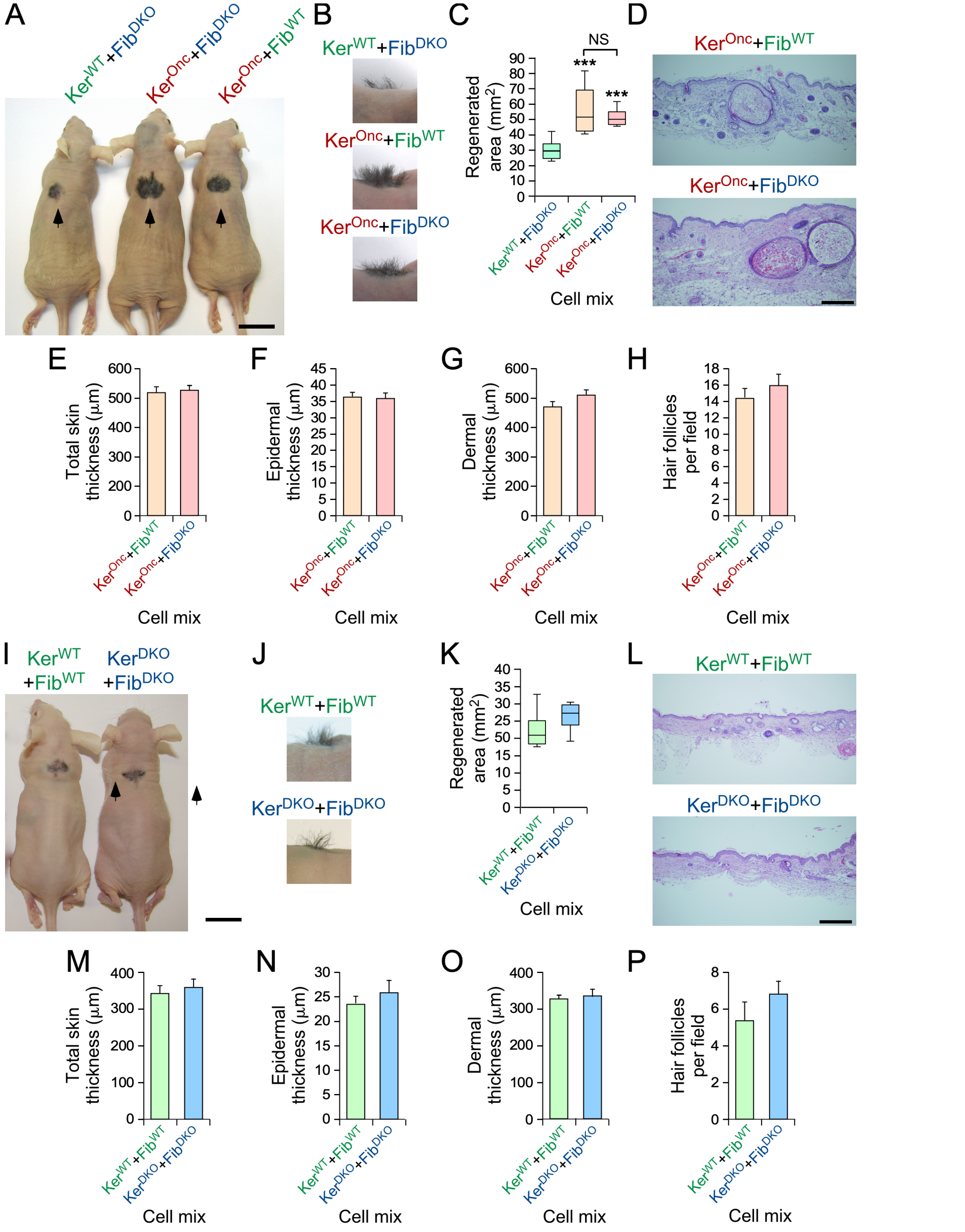

FIGURE S2. The Vav-dependent skin phenotype is mostly keratinocyte-autonomous.

(A) Representative image of immunodeficient mice at the endpoint of the skin xenograft protocol performed with the indicated cell combinations. The regenerated skin area is indicated with an arrow. Scale bar, 2 cm ($n = 4$). DKO, *Vav2*$^{-/-}$;*Vav3*$^{-/-}$ mice. Ker, keratinocytes; Fib, fibroblasts.

(B) Representative images of hair regeneration in immunodeficient mice at the endpoint of the experiment shown in A. $n = 4$ per cell combination.

(C) Quantification of the skin area regenerated by indicated cell type combinations according to the data obtained in A. ***, $P < 0.001$ (ANOVA and Tukey's HSD tests, $n = 4$).

(D) Representative histological sections of the skin area regenerated by the indicated cell mixes at the endpoint of the xenograft protocol. Scale bar, 100 µm (*n* = 4).

(E to **H)** Quantification of the thickness of the skin (E), epidermis (F) and dermis (G) as well as of the number of hair follicles (H) in the histological sections from the experiment shown in D. Differences were not significant (Student's *t*-test, *n* = 4).

(I) Representative image of immunodeficient mice at the endpoint of the skin xenograft protocol performed with the indicated cell type combinations. The regenerated skin area is indicated with an arrow. Scale bar, 2 cm (*n* = 4).

(J) Representative images of hair regeneration in immunodeficient mice at the endpoint of the skin xenograft protocol shown in I and performed with the indicated cell type combinations. *n* = 4 per cell mix.

(K) Quantification of the skin area regenerated by the indicated cell type combinations according to the data obtained in I. Differences were not significant (Student's *t*-test, *n* = 4).

(L) Representative histological sections of the skin area regenerated by the indicated cell mixes at the endpoint of the xenograft protocol. Scale bar, 100 µm (*n* = 4).

(M to P) Quantification of the thickness of the skin (M), epidermis (N) and dermis (O) as well as of the number of hair follicles (P) in the histological sections from the experiment shown in L. Differences were not significant (Student's *t*-test, *n* = 4).

In C, E, F, G, H, K, M, N, O and P, data represent mean ± SEM. NS, not significant.

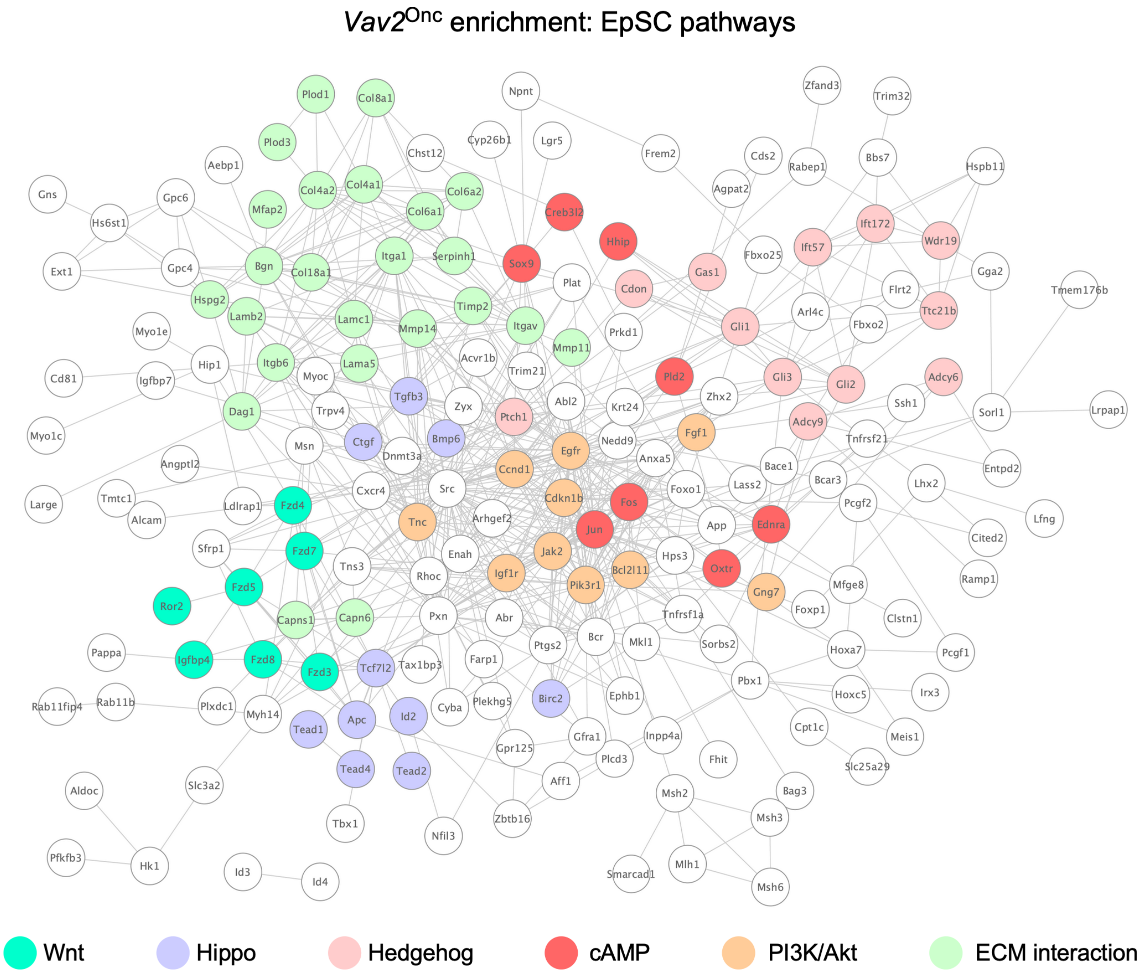

FIGURE S3. Vav proteins regulate transcriptional programs involved in stem cell homeostasis.

Protein-protein interaction network of genes involved in SSC homeostasis that are upregulated by Vav2^{Onc}. Genes associated with the extracellular matrix and the signaling through Wnt, Hippo/Yap, Hedgehog, cAMP and PI3K/Akt pathways are highlighted in color as indicated. ECM, extracellular matrix.

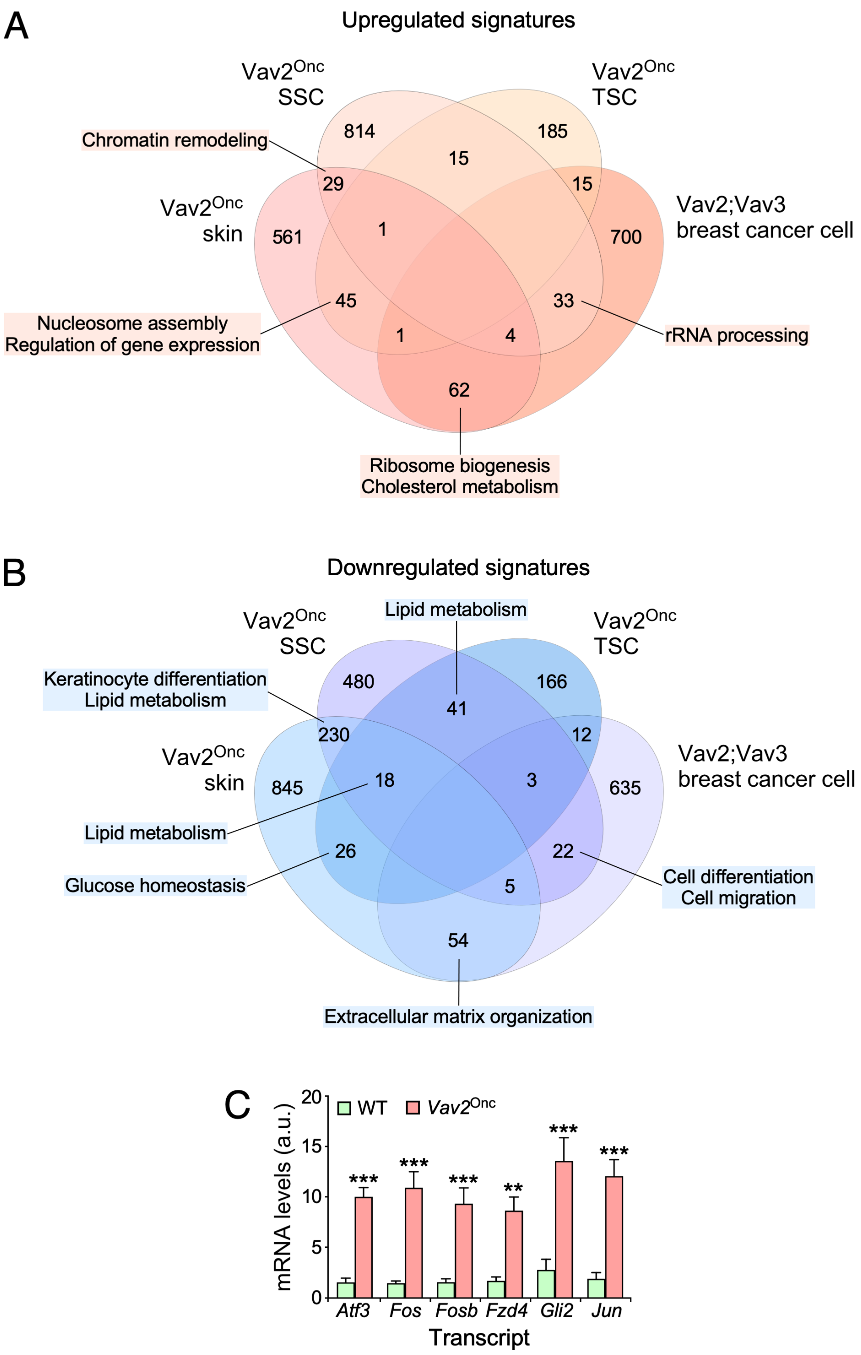

FIGURE S4. Vav2^Onc SSC transcriptome cross-comparison

(A,B) Venn diagrams showing the overlap in upregulated (A) and downregulated (B) genes across the indicated Vav-regulated transcriptional programs. The main functional terms enriched in each of the significant overlapping sets are indicated within colored boxes.

(C) qRT-PCR showing the abundance of interrogated transcripts in SSCs of indicated genotypes. $^{**}P \leq 0.01$, $^{***}P \leq 0.001$ (two-way ANOVA and Sidak's multiple comparisons test, $n = 3$). a.u., arbitrary units. Data represent mean ± SEM.

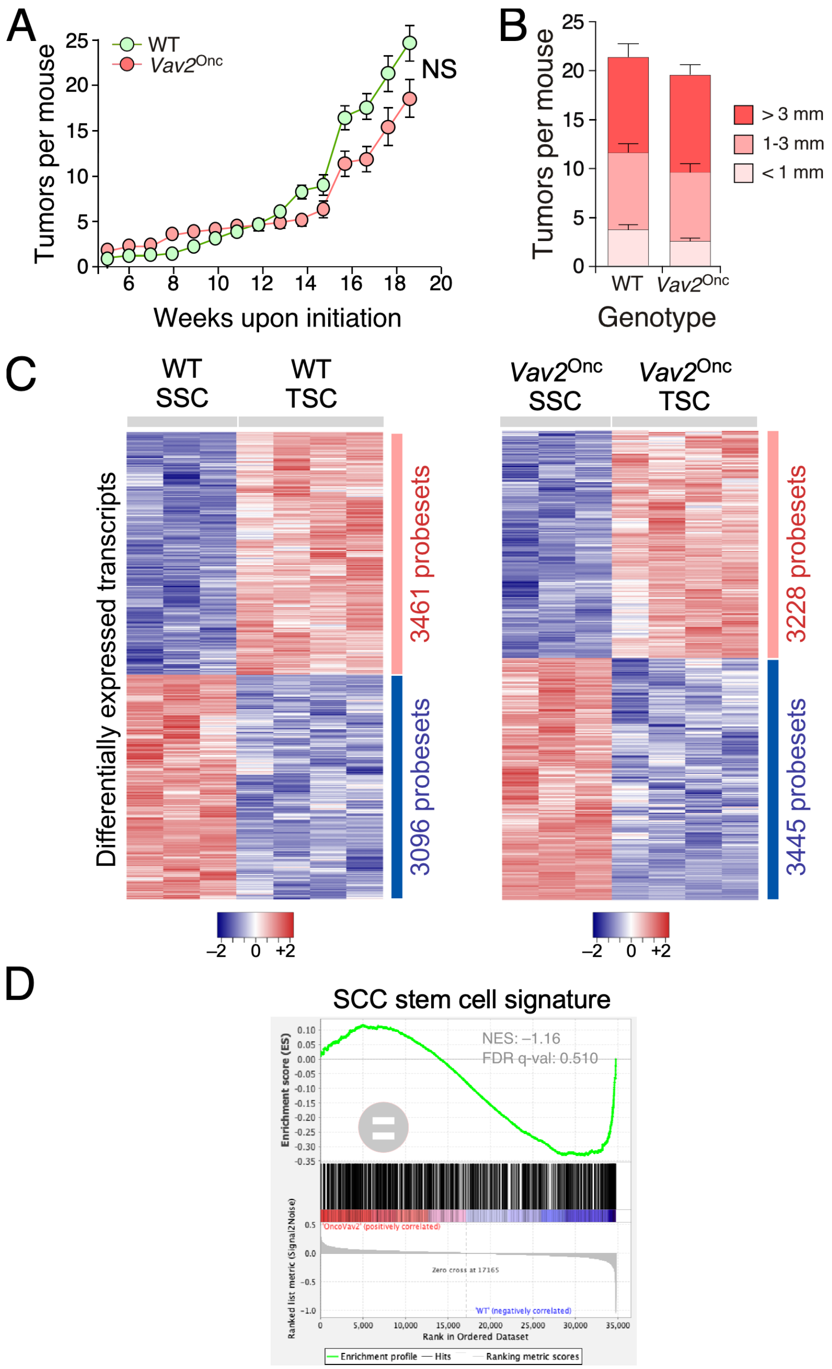

FIGURE S5. Impact of Vav2^{Onc} catalysis-dependent signaling in tumor stem cells.

(A,B) Development kinetics (A) and size (B) of the tumors in DMBA-treated mice of the indicated genotypes. Differences are not significant (NS; Student's *t* test, n = 13 and 15 WT and *Vav2*$^{Onc/Onc}$ mice, respectively). Data represent mean ± SEM

(C) Heatmaps showing the transcripts differentially expressed between TSCs and SSCs in WT (left) *Vav2*$^{Onc/Onc}$ (right) mice. The number of probe sets for the upregulated and downregulated fractions is indicated on the right.

(D) GSEA of indicated gene set in the *Vav2*$^{Onc/Onc}$ TSC transcriptome. The normalized enrichment score (NES) and false discovery rate *q*–value (FDR *q*–val) is indicated inside the graph.
